# Effect of standardized training on the reliability of the Cochrane risk of bias assessment tool: a study protocol

**DOI:** 10.1186/2046-4053-3-144

**Published:** 2014-12-13

**Authors:** Bruno R da Costa, Nina M Resta, Brooke Beckett, Nicholas Israel-Stahre, Alison Diaz, Bradley C Johnston, Matthias Egger, Peter Jüni, Susan Armijo-Olivo

**Affiliations:** Department of Physical Therapy, Florida International University, AHC3-430 11200 8 St, Miami, FL USA; Department of Anesthesia and Pain Medicine, The Hospital for Sick Children, University of Toronto, Toronto, ON Canada; Institute of Health Policy, Management and Evaluation, Dalla Lana School of Public Health, University of Toronto, Toronto, ON Canada; Child Health Evaluative Sciences, The Hospital for Sick Children Research Institute, Toronto, ON Canada; Institute of Social and Preventive Medicine, University of Bern, Bern, Switzerland; Department of Clinical Research, CTU Bern, University of Bern, Bern, Switzerland; CLEAR (Connecting Leadership and Research) Outcomes Research Program, Faculty of Nursing, University of Alberta, Edmonton, AB Canada; Faculty of Rehabilitation Medicine, Department of Physical Therapy, University of Alberta, Edmonton, AB Canada

**Keywords:** Systematic review, Meta-analysis, Risk of bias, RCT, Cochrane

## Abstract

**Background:**

The Cochrane risk of bias (RoB) tool has been widely embraced by the systematic review community, but several studies have reported that its reliability is low. We aim to investigate whether training of raters, including objective and standardized instructions on how to assess risk of bias, can improve the reliability of this tool. We describe the methods that will be used in this investigation and present an intensive standardized training package for risk of bias assessment that could be used by contributors to the Cochrane Collaboration and other reviewers.

**Methods/Design:**

This is a pilot study. We will first perform a systematic literature review to identify randomized clinical trials (RCTs) that will be used for risk of bias assessment. Using the identified RCTs, we will then do a randomized experiment, where raters will be allocated to two different training schemes: minimal training and intensive standardized training. We will calculate the chance-corrected weighted Kappa with 95% confidence intervals to quantify within- and between-group Kappa agreement for each of the domains of the risk of bias tool. To calculate between-group Kappa agreement, we will use risk of bias assessments from pairs of raters after resolution of disagreements. Between-group Kappa agreement will quantify the agreement between the risk of bias assessment of raters in the training groups and the risk of bias assessment of experienced raters. To compare agreement of raters under different training conditions, we will calculate differences between Kappa values with 95% confidence intervals.

**Discussion:**

This study will investigate whether the reliability of the risk of bias tool can be improved by training raters using standardized instructions for risk of bias assessment. One group of inexperienced raters will receive intensive training on risk of bias assessment and the other will receive minimal training. By including a control group with minimal training, we will attempt to mimic what many review authors commonly have to do, that is—conduct risk of bias assessment in RCTs without much formal training or standardized instructions. If our results indicate that an intense standardized training does improve the reliability of the RoB tool, our study is likely to help improve the quality of risk of bias assessments, which is a central component of evidence synthesis.

**Electronic supplementary material:**

The online version of this article (doi:10.1186/2046-4053-3-144) contains supplementary material, which is available to authorized users.

## Background

Systematic reviews and meta-analyses of randomized clinical trials (RCTs) are central to evidence-based clinical decision-making [1, 2]. RCTs are considered the gold standard design when assessing the effectiveness of treatment interventions. Appropriately conducted RCTs may eliminate confounding, allowing decision-makers to infer that changes observed in the outcome of interest are causally linked with the experimental intervention.

If results of RCTs included in a meta-analysis are biased, so will the results of the meta-analysis [3, 4]. To address this problem, it is recommended that the risk of bias in RCTs is taken into consideration when conducting meta-analysis. A method commonly used for this purpose involves the stratification of meta-analyses according to RCTs with low or high risk of bias.

In 2008, the Cochrane Collaboration published a tool and guidelines for the assessment of risk of bias in RCTs [5, 6]. The risk of bias tool has been widely embraced by the systematic review community [7]. The items in this tool address six domains of bias, which are classified as low, high, or unclear risk of bias. The selection of domains of bias was based on empirical evidence and theoretical considerations, focusing on methodological issues that are likely to influence the results of RCTs.

Several studies reported that the reliability of the risk of bias tool is low [8–10]. Reliability of the risk of bias tool can be assessed between two raters of the same research group, when for instance, they assess the risk of bias of RCTs included in a meta-analysis in duplicate. It can also be assessed across research groups, if the risk of bias was assessed for a trial included in two different meta-analyses by two different research groups. Disagreements between two raters of the same research group may be less problematic since they will normally discuss their ratings to come to a consensus. Disagreements between raters from different research groups will be more problematic, for example, if for the same outcome a trial is considered at low risk of bias in one meta-analysis, but is at high risk of bias in another one. Low reliability of risk of bias assessments can then ultimately have repercussions on decision-making and quality of patient care [11, 12].

We recently found that the reliability of the risk of bias tool might be improved by intensive standardized training of raters [8]. However, to our knowledge, no formal evaluation of such a training intervention has been performed. We therefore aim to investigate whether training of raters, with objective and standardized instructions on how to assess risk of bias, can improve the within and between pairs of rater reliability of the Cochrane RoB tool. Here, we describe the methods and the intensive standardized training package for risk of bias assessment that will be used in this study.

## Methods/Design

### Study design

This is a pilot study. The first component is a systematic literature review to identify RCTs that will be used for risk of bias assessment. Using the identified RCTs, the second component of our investigation is a randomized experiment, where raters will be allocated to two different levels of training on risk of bias assessment: minimal training and intensive standardized training.

### Literature search and trial selection

**Figure 1:**
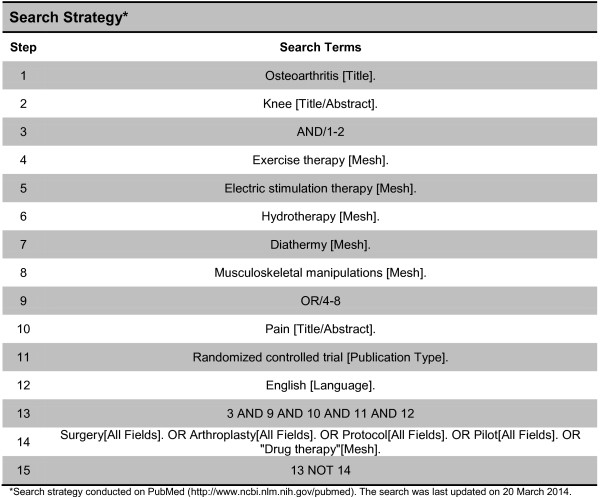
**Search strategy.** *Search strategy conducted on PubMed (http://www.ncbi.nlm.nih.gov/pubmed). The search was last updated on 20 March 2014.

We will search PubMed from inception using database-specific search strategy (Figure 1). We will include every randomized or quasi-randomized clinical trial in patients with knee osteoarthritis that compared a physical therapy intervention to another physical therapy intervention, sham intervention, or no treatment, which assessed patient-reported pain. The following physical therapy interventions will be considered: land-based exercise, aquatic exercise, manual therapy, electric stimulation therapy, and diathermy. We will only consider studies published in English. No further restrictions will be applied. Two raters will screen reports for eligibility independently in duplicate. Disagreements will be resolved by a senior author (BdC).

### Data extraction

We will use standardized, piloted data extraction forms to extract information on publication year, sample size, and type of intervention. We will assess risk of bias for selected items of the risk of bias tool, namely sequence of generation, allocation concealment, blinding (participants, personnel, and assessors), and incomplete outcome data. Although a potentially important source of bias, we will not assess selective outcome reporting in our study due to feasibility issues with such assessment [7]. Within pairs of raters, data extraction will be conducted independently and in duplicate. Potential disagreements within pairs of raters will be solved by discussion until consensus is reached.

### Training on risk of bias assessment

Six raters will assess the risk of bias of every included trial. Four of these raters are doctoral students of physical therapy without previous experience in risk of bias assessment, and two raters are experienced risk of bias assessors. The experienced raters have each been involved in over 15 systematic reviews that included methodological quality assessment. We will use simple randomization (computer-generated numbers http://www.randomizer.org/form.htm) to allocate two students to minimal training and two to intensive training. Randomization will be performed remotely by one of the authors (SAO), who had no contact with the students. Students will not be informed to which training group they have been randomized, and they will be instructed not to discuss their training with each other to minimize the risk of ‘contamination’ [5]. After data extraction is completed, we will ask students to guess in which group they were allocated, whether there was any event during data extraction that made them aware of their group allocation, and if this affected their performance in this study.

Minimal training will mimic assessments made by raters without formal training. It will consist of a single lecture for about 60 min on the definition and importance of each of the assessed domains of bias, without specific or standardized instructions on how to conduct the assessment. In this lecture, the raters will learn how the biases could occur in an RCT (for example, staff recruiting patients who are not concealed to allocation may tamper with random allocation sequences), and they will be shown results of empirical investigations that observed the effect of these biases in clinical treatment effects. The raters will be provided with an article, as an optional reading material, that describes the risk of bias tool [6] as well as chapter 8 of the Cochrane Handbook for Systematic Reviews of Interventions [5], which specifically addresses the assessment of risk of bias of studies included in a systematic review.

Raters allocated to intensive training will receive the same lecture for about 60 min. In addition, they will receive standardized instructions on how to assess each of the domains (Additional file 1). The standardized instructions were based on the Cochrane Handbook [5] and adapted as deemed necessary to increase their objectivity and thus minimize misinterpretations for the assessment of trials of physical therapy in patients with knee osteoarthritis. One of the experienced raters (BdC) will discuss these instructions with them and will give them the opportunity to clarify any questions they may have. This rater is an experienced clinical epidemiologist who was trained by one of the authors of the risk of bias tool (PJ) and has been involved in over 20 systematic reviews that included methodological quality assessment. Next, raters will assess risk of bias in a purposively selected sample of ten articles, which will not be part of the final study sample. One of the experienced raters (BdC) will discuss their assessments after five and ten training articles have been assessed. During these sessions, disagreements between the assessment conducted by the experienced rater and the other raters will be identified. The experienced rater will then provide a rationale to justify his assessment, and discussion will take place until all questions are clarified. The assessments of the raters allocated to intensive training will thus be calibrated with the assessments of the experienced rater.

The raters in both groups will be instructed to not discuss their risk of bias assessment with others.

The study protocol was approved by the research ethics committee of the Florida International University (IRB-14-0110). We will obtain written informed consent from each student rater. This study was not registered in PROSPERO.

### Analysis

We will tabulate the characteristics of included trials and the risk of bias assessments of the three groups of raters (minimal training, intensive training, and experienced raters); before and after consensus. We will then calculate the chance-corrected weighted Kappa with 95% confidence intervals to quantify agreement within and between the three groups of raters for each of the domains of the risk of bias tool. To calculate between-group Kappa agreement, we will use risk of bias assessments from pairs of raters after resolution of disagreements. Kappa values range from 0 to 1, with higher values indicating higher agreement between raters. To calculate the weighted Kappa agreement, risk of bias classification will be ordered as follows: low, unclear, and high risk of bias. Criteria proposed by Byrt [13] will be used to interpret Kappa values. Values between 0.93 and 1.00 represented excellent agreement; 0.81 and 0.92 very good agreement; 0.61 and 0.80 good agreement; 0.41 and 0.60 fair agreement; 0.21 and 0.40 slight agreement, 0.01 and 0.20 poor agreement; and 0.00 or less will be considered to reflect no agreement.

The main outcome of our study is the comparison of the accuracy of assessment across groups, that is, a comparison of the agreement between raters receiving intensive training and experienced raters with the agreement between raters receiving minimal training and experienced raters. To compare agreement of raters under different training conditions, we will calculate the differences between Kappa values for within- and between-group agreement. To address deviations from normal distribution, we will bootstrap the difference in Kappa values using bias correction and acceleration to derive 95% confidence intervals and *P*-values [14]. Please see Additional file 2 for assumptions used for the power analysis.

To explore whether quality of reporting influences agreement, we will stratify the analysis according to publication date (before the first CONSORT statement revision in 2001 vs 2001 [15] and later; and before the latest CONSORT statement revision in 2010 [16] vs 2010 and later), assuming that reporting quality of RCTs in physical therapy improved after the publication of the CONSORT statement [17, 18]. To investigate whether methodological quality influences agreement between raters, we will stratify the analysis by trial size (<100 and ≥100 patients randomized per trial arm), assuming that trial size is associated with methodological quality [19]. A sensitivity analysis will be conducted on larger (≥100 patients randomized per trial arm) trials published after 2010 (i.e. after the latest revision of the CONSORT statement was published). All *P*-values will be two-sided. Analysis will be conducted in STATA, release 13 (StataCorp, College Station, TX).

## Discussion

This study will investigate whether the reliability of the risk of bias tool can be improved by training raters using standardized instructions for risk of bias assessment. We will calculate Kappa coefficients to quantify the agreement between experienced raters and inexperienced raters. Two inexperienced raters will receive intensive training on risk of bias assessment and the other two will receive minimal training. By including a control group with minimal training, we will attempt to mimic what many review authors commonly have to do, that is—conduct risk of bias assessments of RCTs without any formal training or standardized instructions. If our results indicate that an intensive standardized training does improve the reliability of the risk of bias tool, our study is likely to help improve the quality of risk of bias assessment, which is a central component of evidence synthesis [4]. The standardized training package for risk of bias assessment could then be used by organizations such as the Cochrane Collaboration and the systematic review community at large. By publishing this protocol, we would also like to make the objectives and pre-specified study design transparent to readers, as has been recommended for methodological studies [20].

### Strengths and limitations

Our study had two major strengths. First, we will include raters completely inexperienced with the risk of bias assessment to investigate the effect of intensive training on the reliability of the risk of bias tool. By including inexperienced raters, we believe we will be more likely to observe an effect of intensive training, if one indeed exists. If raters were already experienced with the risk of bias assessment, there could be limited room for improvement as postulated in a previous study that investigated the effect of training on a similar method for methodological quality assessment [21]. Second, raters will be randomly allocated to training groups, and central randomization will be performed to conceal the random sequence of allocation. We acknowledge that this approach may not create a balanced distribution of potential confounders across the two training groups due to the low number of individuals randomized, but the group of students randomized will be fairly homogeneous and confounding therefore unlikely.

Our results can potentially be influenced by performance bias. If raters in the control group understand that they are not receiving the best training available in our study, they may feel discouraged to try and perform risk of bias assessments as best as they can. This could in turn lead to an artificially lower reliability of the risk of bias tool with minimal training as compared to intensive training. Alternatively, they could seek additional training elsewhere or be prompted to self-study. To try and minimize the risk of such performance bias, raters will not be informed to which training group they have been randomized, and they will be instructed to not discuss with each other any characteristics of their training. The use of minimal training as a control intervention may lead to an underestimation of the effect of our intensive training, however. Although ‘no training’ could be used as a control intervention instead of minimal training, this could substantially increase the risk of performance bias in our study, as explained above.

Although the standardized instructions were primarily based on the Cochrane Handbook instructions for risk of bias assessment, we added or adapted some of the criteria to facilitate raters’ decision-making in the present investigation. Some of these criteria are not solely based on empirical evidence but also on our own experience with analyses of individual RCTs and meta-analyses of RCTs (Additional file 1). The main example concerns the assessment of incomplete outcome data. To facilitate decision-making, we used thresholds of drop-out rates to define low and high risk of bias for assessing incomplete data. These thresholds were chosen based on evidence that this drop-out rate could be linked to biases [22, 23]. Moreover, some of these adaptations may not apply to systematic reviews with different research questions. For instance, because outcomes in our sample of physical therapy trials could potentially be influenced by at least some degree of performance bias, blinding of patients and therapists were by definition deemed necessary. Conversely, the Cochrane Handbook recommends that raters first assess the need for patient and therapist blinding in each trial in light of the outcome of interest and to take this information into consideration when assessing the risk of performance bias. Although we fully agree with this recommendation, it was not included in our standardized instructions for the reason explained above.

The low number of raters randomized to intervention groups will be a limitation for the generalizability of our findings. Due to feasibility issues, we included the minimal number of participants needed to calculate Kappa agreements within each study condition. If results of the present investigation indicate that intensive training may indeed improve the reliability of the risk of bias tool, a future study including a larger number of raters could replicate our methods to address this limitation. Generalizability may be further hindered by the characteristics of the trials assessed in our study and of the individual providing the training on risk of bias assessment. Accuracy of the risk of bias assessment could vary if trials with different patient populations, interventions, and outcomes were assessed. Likewise, the quality of the training on the risk of bias assessment may vary according to the skills of the person who provides the training and the method used to deliver such training.

### Previous research

The risk of bias tool has been extensively used in many Cochrane reviews, albeit the information of the inter-rater reliability of the risk of bias tool is rather limited. To date, five studies [9, 10, 24–26] have investigated the inter-rater reliability of the risk of bias tool, but none have proposed and investigated ways to improve its reliability. The inter-rater agreement for the individual domains of the risk of bias tool has been found to range from poor (Kappa = 0.13 for selective reporting) to substantial (Kappa = 0.74 for sequence generation) [25]. Our team recently investigated the between-group Kappa agreement of the risk of bias tool comparing ratings from Cochrane reviewers to ratings from our team [8]. Most of our Kappa values were considerably higher than those reported in previous studies. We suspect two key factors may explain these differences. Although we used the Cochrane Handbook guidelines for risk of bias assessments, we first predefined specific decision rules to assess the individual domains of the tool. Second, we used an intensive training for the raters to standardize the decision-making process. This could partly explain the results of our previous study. The current study will investigate this hypothesis.

## Electronic supplementary material

Additional file 1: **Guidelines for evaluating the risk of bias in physical therapy trials with patients with knee osteoarthritis.** (DOC 72 KB)

Additional file 2: **Assumptions of the power analysis.** (DOCX 13 KB)

## Abbreviations

*RoB*: risk of bias
*RCT*: randomized clinical trial
*PROSPERO*: international prospective register of systematic reviews
*CONSORT*: consolidated standards of reporting trials.
