# Supplementary material for: Effect of standardized training on the reliability of the Cochrane risk of bias assessment tool: a study protocol
Source: Syst Rev. 2014 Dec 13;3:144. doi: 10.1186/2046-4053-3-144 (PMC4273317; doi:10.1186/2046-4053-3-144)
Supplement: Supplementary file 1 — Additional file 1: Guidelines for evaluating the risk of bias in physical therapy trials with patients with knee osteoarthritis. (DOC 72 KB) [file 13643_2014_310_MOESM1_ESM.doc]

**Guidelines for evaluating the risk of bias in physical therapy trials with patients with knee osteoarthritis.**

| **RANDOM SEQUENCE GENERATION** | |
| --- | --- |
| Criteria for a judgement of  **‘Low risk’ of bias.** | The investigators describe a random component in the sequence generation process such as:   - Random number table; - Computer random number generator; - Coin tossing; - Shuffling cards or envelopes; - Throwing dice; - Drawing of lots; - Minimization*.     *Minimization may be implemented without a random element, and this is considered to be equivalent to being random. |
| Criteria for the judgement of  ‘**High risk’ of bias.^b^** | The investigators describe a non-random component in the sequence generation process. Usually, the description would involve some systematic, non-random approach, for example:   - Date of birth; - Date (or day) of admission; - Hospital or clinic record number. - Alternation (i.e. first patients receives treatment A, second patients receives treatment B, and so on)^a^ |
| Criteria for the judgement of  **‘Unclear risk’ of bias.** | Insufficient information about the sequence generation process to permit judgement of ‘Low risk’ or ‘High risk’. |
| **ALLOCATION CONCEALMENT** | |
| Criteria for a judgement of  **‘Low risk’ of bias.** | Participants and investigators enrolling participants could not foresee assignment because one of the following, or an equivalent method, was used to conceal allocation:   - Central allocation (including telephone/fax, web-based and pharmacy-controlled randomization); - On-site computer system^a^ - Sequentially numbered drug containers of identical appearance; - Sequentially numbered, opaque, sealed envelopes (all 3 characteristics need to be present) |
| Criteria for the judgement of  **‘High risk’ of bias.** | Participants or investigators enrolling participants could possibly foresee assignments and thus introduce selection bias. Examples of inappropriate methods of allocation:   - Open random allocation schedule; - Assignment envelopes were used without appropriate safeguards (e.g. if envelopes were unsealed or non­opaque or not sequentially numbered); - Alternation or rotation; - Date of birth; - Case record number; - Any other predictable or explicitly unconcealed procedure. |
| Criteria for the judgement of  **‘Unclear risk’ of bias.** | Insufficient information to permit judgement of ‘Low risk’ or ‘High risk’. This is usually the case if the method of concealment is not described or not described in sufficient detail to allow a definite judgement – for example if the use of assignment envelopes is described, but it remains unclear whether envelopes were sequentially numbered, opaque or sealed. (Not all of 3 characteristics are present). |
| **BLINDING OF PARTICIPANTS AND PERSONNEL**   - Assess blinding of patients and blinding of study personnel separately^a^ | |
| Criteria for a judgement of  **‘Low risk’ of bias.^b^** | Any one of the following:   - Participants and study personnel explicitly described as ‘blinded’, and unlikely that the blinding could have been broken. If only "investigators" are explicitly described as blinded, assume that these include study personnel |
| Criteria for the judgement of  **‘High risk’ of bias.^b^** | Any one of the following:   - No blinding (‘open’ or ‘non-blinded’) or incomplete blinding; - Blinding of study participants and personnel attempted, but likely that the blinding could have been broken. |
| Criteria for the judgement of  **‘Unclear risk’ of bias.** | Any one of the following:   - Insufficient information to permit judgement of ‘Low risk’ or ‘High risk’; - The study did not address the issue of blinding. |
| **BLINDING OF OUTCOME ASSESSMENT**   - Only assess the primary outcome. If primary outcome is not clearly specified, then assess the first outcome reported in the results section.^c^ - Blinding of participants/patients must take place when outcomes are patient-reported.^a^ - When outcomes are measured by an assessor other than the patients themselves, then assessors should be blinded to group allocation if outcomes are subjective (i.e. outcomes are likely to be influenced by lack of blinding)^a^ | |
| Criteria for a judgement of  **‘Low risk’ of bias.^b^** | Any one of the following:   - Outcome assessors explicitly described as ‘blinded’, and unlikely that the blinding could have been broken. If only "investigators" are explicitly described as blinded, assume that these include outcome assessors; - No blinding of outcome assessment, but the outcome is objective and therefore not likely to be influenced by lack of blinding. |
| Criteria for the judgement of  **‘High risk’ of bias.** | Any one of the following:   - No blinding of outcome assessment (‘open’ or ‘non-blinded’), and the outcome assessment is likely to be influenced by lack of blinding; - Blinding of outcome assessment, but likely that the blinding could have been broken and the outcome assessment is likely to be influenced by lack of blinding. |
| Criteria for the judgement of  **‘Unclear risk’ of bias.** | Any one of the following:   - Insufficient information to allow judgement of ‘Low risk’ or ‘High risk’; - The study did not address the issue of blinding. |
| **INCOMPLETE OUTCOME DATA**   - Only assess the primary outcome. If primary outcome is not clearly specified, then assess the first outcome reported in the results section. If time point for primary outcome is not specified, look at the last assessment reported.^c^ - If authors claimed that an ITT analysis was performed, raters (YOU) should confirm that all patients entered were accounted for in the analysis according to randomization (i.e., do not assume that a true ITT analysis was done and make sure that the number of patients randomized per group match the number of patients analyzed in each group; beware of modified ITT analyses).^a^ | |
| Criteria for a judgement of  **‘Low risk’ of bias.^b^** | Any one of the following:   - No missing outcome data (If all patients were accounted for in the analysis) - If the numbers and reasons for withdrawal/drop-out were described and comparable across groups **and the drop out rate was ≤10% drop outs, then score low risk of bias^a^** - If the numbers and reasons for withdrawal/drop-out were described and comparable across groups **and the authors performed an ITT analysis (multiple imputation) with ≤ 20% drop outs, then score low risk of bias^a^** |
| Criteria for the judgement of  **‘High risk’ of bias.^b^** | Any one of the following:   - Numbers and reasons for withdrawal/drop-out were described and are NOT comparable across groups; - Potentially inappropriate application of simple imputation, such as ‘last-observation-carried-forward’ with >10% and ≤ 20% drop outs^a^ - **> 20% drop outs^a^** |
| Criteria for the judgement of  **‘Unclear risk’ of bias.^b^** | Any one of the following:   - Insufficient reporting of attrition/exclusions to permit judgement of ‘Low risk’ or ‘High risk’ (e.g. number of patients randomized not reported, no reasons for missing data provided); - **No ITT but >10% and ≤ 20% drop outs^a^** |

**^a^Not listed in the Cochrane Handbook**

**^b^Different from the Cochrane Hanbook**

**^c^The Cochrane Handbook recommends that assessment should be conducted for all outcomes of interest to a particular systematic review. In the present study, we only assessed the primary outcome of each trial.**
