# Supplementary material for: Effect of standardized training on the reliability of the Cochrane risk of bias assessment tool: a study protocol
Source: Syst Rev. 2014 Dec 13;3:144. doi: 10.1186/2046-4053-3-144 (PMC4273317; doi:10.1186/2046-4053-3-144)
Supplement: Supplementary file 2 — Additional file 2: Assumptions of the power analysis. (DOCX 13 KB) [file 13643_2014_310_MOESM2_ESM.docx]

**Additional file 2** Assumptions of the power analysis.

We estimate that raters will assess a convenience sample of approximately 60 RCTs (all RCTs of physical therapy for knee osteoarthritis ever published that reported pain as an outcome). We conducted a simulation exercise using STATA that indicated that this number of RCTs would give us approximately 80% power for the comparison of the Kappa agreement from experienced raters and intensive training raters (Kappa 1), with the Kappa agreement from experienced raters and minimal training raters (Kappa 2), considering the following parameters: Kappa 1 = 0.75 (good agreement), Kappa 2 = 0.45 (fair agreement), alpha = 0.05.
